# The Role of B and T Lymphocyte Attenuator in Respiratory System Diseases

**DOI:** 10.3389/fimmu.2021.635623

**Published:** 2021-06-07

**Authors:** Zheng Deng, Yi Zheng, Pei Cai, Zheng Zheng

**Affiliations:** ^1^ General Department, Hunan Institute for Tuberculosis Control, Changsha, China; ^2^ General Department, Hunan Chest Hospital, Changsha, China; ^3^ Department of Pharmacy, Hunan Provincial Maternal and Child Health Care Hospital, Changsha, China

**Keywords:** BTLA, airway inflammation, lung cancer, asthma, sepsis

## Abstract

B and T lymphocyte attenuator (BTLA), an immunomodulatory molecule widely expressed on the surface of immune cells, can influence various signaling pathways and negatively regulate the activation and proliferation of immune cells by binding to its ligand herpes virus entry mediator (HVEM). BTLA plays an important role in immunoregulation and is involved in the pathogenesis of various respiratory diseases, including airway inflammation, asthma, infection, pneumonia, acute respiratory distress syndrome and lung cancer. In recent years, some studies have found that BTLA also has played a positive regulatory effect on immunity system in the occurrence and development of respiratory diseases. Since severe pulmonary infection is a risk factor for sepsis, this review also summarized the new findings on the role of BTLA in sepsis.

## Introduction

Nowadays, more and more studies focus on the immunological pathogenesis of diseases. Immune dysfunction plays a pivotal role in the development of inflammation, infection and tumor. Many immunomodulatory chemicals and targeted drugs have been used in clinical practice. As a cosignaling molecule, B and T lymphocyte attenuator (BTLA) plays an important role in immunoregulation and is involved in the pathogenesis of various respiratory diseases. In this review, we discuss the biological characteristics of BTLA and explore their role in respiratory diseases.

## The Biological Function of BTLA

BTLA, a co-inhibitory molecule similar to cytotoxic T lymphocyte antigen-4 (CTLA-4) and programmed death 1 (PD-1), belongs to the immunoglobulin superfamily, and mainly negatively regulates the activation and proliferation of immune cells (1, 2). BTLA is widely expressed on the surface of various immune cells, such as B cells, T cells, monocytes, macrophages, dendritic cells (DCs), natural killer cells (2, 3). At all phases of T cell differentiation including naive T cell, BTLA expression exists (4, 5). BTLA is a transmembrane glycoprotein. Its extracellular domain is immunoglobulin domain, and intracellular domain contains three conserved motifs: one is the proximal motif which have sequence YDND, an immunoreceptor tyrosine-based inhibitory motif (ITIM), and an immunoreceptor tyrosine-based switch motif (ITSM) (1, 6, 7). The motif of sequence YDND contains tyrosine and binds to growth factor receptor binding 2 (Grb-2), then interacting with the p85 subunit of PI3K, which is related to the pro-survival function of BTLA (6, 8). The ITIM phosphorylate after BTLA binds to HVEM. Then ITIM recruit and activate protein tyrosine phosphatases SHP-1 and SHP-2, attenuating tyrosine kinase activated by T cell receptor, thus leading to inhibitory effects (1, 4, 8). A research showed that either ITIM or ITSM mutation abolished the interaction between SHP-1 and SHP-2 with BTLA, indicating that ITSM is involved in SHP-1 and SHP-2 activation along with ITIM (8).

Herpes virus entry mediator (HVEM), which belongs to tumor necrosis factor receptor (TNFR) superfamily, was identified to be the only ligand of BTLA. It is also widely expressed on the surface of immune cells like T cells, B cells and dendritic cells (9). Additionally, HVEM is also expressed on non-immune cell including intestine and lung epithelial cells and is essential for host protection as an innate immune mediator when stimulated by BTLA-Ig (10). BTLA and HVEM can even be co-expressed on T cells and form a cis-complex, which can help T cells stay in the naive state, thus inhibiting T cell proliferation and differentiation and modulating immune homeostasis (11). BTLA significantly weakens the activation of transcription factor NF-κB and NFAT, inhibits the activity of transcription factor AP-1, thus inhibiting the proliferation and differentiation of T cells (12). Besides the inhibition role of BTLA to T cell proliferation, BTLA can also prevent Toll-like receptors in dendritic cells from being over-activated, reduce cytokine production of NKT cells and follicular Th cells (13–16). *BTLA* gene is also regulated by other factors. The transcriptional coactivator Bob1 along with the octamer transcription factors Oct1/Oct2 can directly bind to and transactivate the promoters of BTLA. Bob1 is required for the expression of normal levels of BTLA (17). In γδ T cells, transcription factor: retinoid-related orphan receptor gamma-t (RORγt) can inhibit the transcription of BTLA, and IL-7 can raise the level of BTLA protein on the surface of cells (18).

## BTLA in Airway Inflammation and Asthma

Allergic airway inflammation can cause immune response in the lung, which is mediated by Th2 cells and their cytokines such as IL-4, IL-5, and IL-13, and subsequently cause epithelial damage and airway hyperreactivity (19). As an immunoregulatory molecule, BTLA is involved in the development of allergic airway inflammation (**Table 1**). Deppong C et al. found that after allergens inhalation, the airway inflammation of the sensitized wild type mice reached to its peak by day 3, and resolved by day 10, while the airway inflammation of the mice deficient in expressing BTLA could last as long as 15 days, indicating that BTLA can shorten the duration of allergic airway inflammation and give proper termination to the acute inflammatory response (20). Another research focused on the role of BTLA in the proliferation, recruitment, and survival of T cells in response to inhaled allergens in BTLA-deficient mice and wild type mice. Decreased cell death of T cells was found in BTLA deficient mice, whereas proliferation and recruitment of T cells to the lungs remained unaffected, indicating BTLA signaling is a key determinant of the inflammatory response in the lung (22). Apart from that, BTLA is involved in suppression of the induction of allergic airway inflammation. Tamachi et al. found IL-5 production and eosinophilic inflammation were increased in the airways of BTLA deficient mice after the antigen inhalation (21). The above research results showed that BTLA could regulate the death of T cells in lung and inhibit the aggregation of eosinophils induced by endobronchial antigens. However, the precise mechanism is still unclear and no information on the role of BTLA in allergic diseases in humans is available. Further analysis is needed to reveal the role of BTLA in the biologic basis of eosinophil-mediated allergic diseases. Considering that asthma is associated with intrabronchial aggregation of eosinophils and disproportion of T cell subsets, BTLA may be correlated with the pathogenesis of asthma. A research with children found that the variation of multi-loci on BTLA gene could influence serum IgE levels (23), which indirectly indicated that BTLA might be associated with asthma. These studies indicated that BTLA may serve as a novel target for the therapeutic intervention.

**Table 1:** The function of BTLA in acute allergic airway inflammation.

Study. Year	Specimen	Models	Main findings	Reference
Deppong et al., 2006	BALF	BTLA^–/–^ mice WT mice	BTLA are required for the termination of acute allergic airway inflammation.	(20)
Tamachi et al., 2007	Spleen BALF	BTLA^–/–^ mice WT mice	BTLA inhibits antigen-induced eosinophil recruitment into the airways by preventing IL-5 production from Th2 cells.	(21)
Deppong et al., 2008	Spleen BALF Lung	BTLA^–/–^ mice WT mice	BTLA limits the duration of airway inflammation following both single and repeated allergen challenges.	(22)

BALF, bronchoalveolar lavage fluid; BTLA, B and T lymphocyte attenuator; BTLA^–/–^, BTLA-deficient; WT, wild-type.

## BTLA and Infection

Antigen-specific T cells are crucial for the anti-infective effect. Recent studies have shown that BTLA also plays a role in immune responses against infectious pathogens. Since BTLA is widely expressed on the surface of immune cells, its relation to immune response of infection including innate immune and adaptive immune has been a hot topic. A research indicated that BTLA was critical for negatively regulating early host immunity against intracellular bacteria. Compared with wild type mice, HVEM and BTLA deficient mice were more resistant to listeriosis. Blocking BTLA signaling pathway could promote early removal of bacteria. Stimulated by the Listeria, innate immune cells of BTLA deficient mice secreted significantly more proinflammatory factors, which indicated that BTLA played an important regulatory role in early host innate immune response against infection (24). BTLA also negatively regulates immune response to virus. Cytomegalovirus (CMV) infection could induce high expression of BTLA on virus specific CD8^+^ T cells. Using antibody to block BTLA *in vitro* could facilitate the proliferation of virus specific CD8^+^ T cells (25). On the contrary, some studies found that the expression of BTLA help fight against infection. Marcos W. Steinberg et al. found that in BTLA and HVEM deficient mice and mice with an BTLA-HVEM blockade, the number of antigen specific CD8^+^ T cells was reduced after bacterial infection. This result suggests that BTLA-HVEM signaling pathway does not restrict to inhibitory signaling transmission, BTLA can promote the survival of antigen specific CD8^+^ T cell to fight against bacterial infection through HVEM dependent signal pathway (26). Similar results were also found in viral infection. The numbers of the effector CD8^+^ T cells and memory CD8^+^ T cells were reduced after BTLA or HVEM deficient mice infected with vaccinia virus. HVEM-BTLA signaling could promote the differentiation of memory CD8^+^ T cells to defend viral infection (27). The positive effect of the signaling pathway may be related to *tran*-interaction of BTLA with HVEM, while *cis*-interaction shows negative effect. Coronavirus disease 2019 (COVID-19), which is caused by the severe acute respiratory syndrome coronavirus 2 (SARS-CoV-2), is globally pandemic. There were some researches showing the relation between BTLA and COVID-19. Both Christoph Schultheiß et al. and Narjes Saheb Sharif-Askari et al. found that BTLA was upregulated compared to that in controls (28, 29). Marissa Herrmann et al. found that the level of BTLA on CD8^+^ T cell decreased in COVID-19, but not as strong as in healthy controls, and the expression of BTLA on transitional memory and effector memory CD8^+^ T cells in COVID-19 was higher compared to healthy controls (30).

The researches on the role of BTLA in pulmonary infection mainly focused on tuberculosis (TB) infection (**Table 2**). The chronic infection of Mycobacterium tuberculosis (Mtb) indicates the protective (escaping) strategies to avoid clearance by the innate and adaptive immune responses (36). Shen et al. found that BTLA was upregulated on circulating CD4^+^ and CD8^+^ T cells of pulmonary TB patients. The level of BTLA expression was dynamically changed with the increase of TB bacillary load, suggesting that BTLA could be used as a useful marker reflecting immune function as well as disease progression (33). Wang et al. analyzed the role of BTLA in antigen presenting cells (APCs) and found that BTLA was highly expressed in CD11c-expressing APCs in patients with active pulmonary tuberculosis (ATB). The BTLA-expressing CD11c APCs showed decreased capacity to stimulate allogeneic T cell proliferation which was associated with low expression of HLA-DR and less IL-6 secretion in ATB patients (32). An extension study showed that TB-driven BTLA expression in DCs could affect their biological characteristics and immune functions, which was associated with an increased capacity to produce IL-4 and TGF-β and a decreased capacity of DCs to produce the key cytokine IL-12, and to induce T cell proliferation and differentiation into Th subsets, resulting in altered anti-TB immune responses and immunity (35). An analogous finding showed that high co-expression of BTLA and B7-H4 on myeloid dendritic cells (mDCs) in peripheral blood and pleural effusions of pleural TB patients promoted a high level of CD83 and HLA-DR, which had a negative regulatory effect on mDCs and anti-TB immunity (34). In contrast to the up-regulation of BTLA expression in circulating CD4^+^ and CD8^+^ T cells, APCs, and DCs, the expression of BTLA was decreased in αβ T cells of active pulmonary tuberculosis patients and anti-tuberculosis drugs induced BTLA expression along with bacterial clearance. BTLA expression on αβ T cells was associated with protective immune memory in ATB patients against Mtb infection (31). Unlike the role of BTLA in negative regulation of immune responses, this result indicates that BTLA is involved in pathogen clearance.

**Table 2:** The function of BTLA in tuberculosis.

Study. Year	Specimen	Immune cells	Subjects	Main findings	Reference
Zeng et al., 2014	Peripheral blood samples	αβ T cells	68 ATB patients 40 healthy controls	1. BTLA expression on αβ T cells is decreased in ATB patients. 2. BTLA expression on αβ T cells is likely associated with protective immune memory against mycobacterium tuberculosis infection.	(31)
Wang et al., 2016	Peripheral blood samples	CD11c APCs	52 ATB patients 15 healthy controls	1. The frequencies of BTLA positive CD11c APCs in ATB patients were higher than that in healthy controls. 2. BTLA-expressing CD11c APCs in ATB patients show low capacity to stimulate T cell proliferation.	(32)
Shen et al., 2019	Peripheral blood samples	CD4^+^ and CD8^+^ T cells	86 ATB patients 40 healthy controls	The levels of BTLA expression were upregulated on peripheral CD4^+^ and CD8^+^ T cells of ATB patients and associated with disease progression.	(33)
Cai et al., 2019	Peripheral blood samples Pleural effusions	mDCs	20 tuberculous pleurisy patients 15 healthy controls	1. Co-expression of BTLA and B7-H4 on myeloid dendritic cells DCs (mDCs) in peripheral blood and pleural effusions of pleural TB patients was significantly higher than in the control group. 2. High expression of BTLA and B7-H4 promoted a high level of CD83 and HLA-DR, which had a negative regulatory effect on mDCs on anti-TB immunity.	(34)
Zhang et al.2020	Peripheral blood samples	mDCs and pDCs	73 ATB patients 35 healthy controls	1. ATB patients exhibited higher expression of BTLA in mDCs and pDCs subsets than healthy controls. 2. TB-driven BTLA expression in DCs impairs the expression of functional DC surrogate markers and suppress the ability of DCs to induce anti-TB Th17 and Th22 response while promoting Th2 and Foxp3+ Tregs.	(35)

APCs, antigen presenting cells; ATB, active pulmonary tuberculosis; BTLA, B and T lymphocyte attenuator; mDCs, myeloid dendritic cells; Mtb, mycobacterium tuberculosis; pDCs, plasmacytoid DCs; TB, tuberculosis.

## BTLA and Sepsis

Inflammatory responses play a critical role in the pathogenesis of pneumonia, and the intensity of these responses often determines the severity of the disease. Severe pulmonary infection is a risk factor for sepsis. BTLA signaling can induce several immune responses such as immune tolerance, immunosuppression, and immune escape. Previous researches have demonstrated that BTLA plays a role in regulating the immune response in sepsis (**Table 3**). Nicholas J. Shubin et al. found that the number of BTLA and HVEM expressing macrophages, dendritic cells, neutrophils increased in the original infection site of septic mice. BTLA deficient septic mice showed higher survival rate than wild type septic mice (37). Another research showed similar results that BTLA could suppress LPS induced endotoxic shock by suppressing cytokine production from LPS-stimulated dendritic cells and macrophages (14). However, Cheng et al. found different phenomena. Treating septic mice with anti-BTLA antibody, cytokines and inflammatory cells increased in the original site of infection, and the mice exhibited more severe organ impairment and lower survival rate (40). Differences also exist among clinical researches. A research found that the level of soluble BTLA in the serum of septic patients was much higher than that of ICU non-septic control and healthy control, and the level was associated with Sequential Organ Failure Assessment (SOFA) score, which is calculated by various indicators such as the oxygenation index, the Glasgow coma scale, the level of platelet, creatinine and so on. The level of sBTLA in 28 days sepsis non-survivors was significantly higher than in survivors (41). Similar result was found by Sean F. Monaghan et al., and sBTLA can predict the diagnosis of sepsis (43). Nicholas J Shubin et al. exhibited that in the peripheral blood from ICU patients with sepsis, the proportion of BTLA expressing CD4^+^ T cells increased. In critically ill patients without sepsis, if over 80% of the CD4^+^ T cells expressed BTLA, they developed nosocomial infections more easily and had longer hospital stays (38). The BTLA density on the surface of peripheral blood CD4^+^ T cells was upregulated in sepsis survivors compared to healthy controls (42). Differently, Rui Shao et al. found that in severe sepsis and septic shock patients, the proportion of peripheral blood BTLA^+^/CD4^+^ T cells was significantly reduced compared with healthy volunteers, and that ratio was lower in septic non-survivors compared to septic survivors (39). The differences among above researches may be due to the timing of entry points. The expression of BTLA at different stage of sepsis may have different clinical effects. At the early stage of sepsis, which is the proinflammatory stage, the expression of BTLA may increase along with the enhanced inflammatory reaction, so as to protect organs from inflammatory storm. While at the anti-inflammatory stage, high expression of BTLA inhibits the activation of immune cells, and excessive immune suppression may lead to a secondary infection and bad prognosis.

**Table 3:** The function of BTLA in sepsis.

Study. Year	Specimen	Subjects	Main findings	Reference
Shubin et al. 2012	Peripheral blood Peritoneal lavage fluid	BTLA^–/–^ mice WT mice 20 sepsis patients 7 nonseptic, critically ill patients	1. The number of infiltrating BTLA- and HVEM-expressing macrophages, inflammatory monocytes, mature and immature DCs, and neutrophils increased in the peritoneum in mice with acute experimental sepsis induction. 2. BTLA and HVEM monocytes in peripheral blood and HVEM granulocytes were increased in septic ICU patients. 3. BTLA can serve as makers to predict the occurrence of sepsis.	(37)
Shubin et al. 2013	Peripheral blood Spleen Thymus	BTLA^–/–^ mice WT mice 39 critically ill ICU patients 6 healthy controls	1. The septic ICU patients had a higher percentage of BTLA^+^ CD4^+^ lymphocytes in peripheral blood compared with critically ill non-septic individuals. 2. BTLA expression in circulating CD4^+^ T-cell and B-cell increased in septic mice. 3. Ill patients with CD4^+^ T-cells expressing greater than 80% BTLA^+^ had longer hospital stays.	(38)
Shao et al. 2015	Peripheral blood	286 sepsis patients 50 healthy controls	1. BTLA^+^/CD4^+^ T cells was high expressed in healthy volunteers and was reduced in severe sepsis and septic shock patients. 2. The percentage of BTLA^+^/CD4^+^T cells was lower in non-survivors than that in survivors.	(39)
Cheng et al. 2016	Peripheral blood Peritoneal lavage fluid	Sepsis mice model	1. BTLA expression is elevated on innate immune cells in mice model of hemorrhagic shock/sepsis. 2. Anti BTLA antibody treatment increased cytokine/chemokine levels and inflammatory cells recruitment, aggravated organ injury and elevated these animals’ mortality.	(40)
Lange et al. 2016	Peripheral blood	101 patients with sepsis 28 ICU controls 31 healthy controls	Soluble BTLA levels in plasma were higher in the sepsis cohort and is associated with severity of disease.	(41)
Arens et al. 2016	Peripheral blood	8 sepsis patients 8 healthy controls	BTLA was upregulated in CD4^+^ cells of sepsis survivors.	(42)
Kobayashi et al. 2018	Spleen Bone marrow	BTLA^–/–^ mice WT mice	1. BTLA^–/–^ mice are more susceptible to LPS-induced endotoxic shock. 2. BTLA inhibit LPS-induced cytokine production in dendritic cells and macrophages. 3. Anti-BTLA antibody save mice from LPS-induced endotoxic shock.	(14)

BTLA, B and T lymphocyte attenuator; BTLA^–/–^, BTLA-deficient; WT, wild-type; LPS, lipopolysaccharide.

## BTLA in Pneumonia and Acute Respiratory Distress Syndrome

Inflammatory responses are involved in the immunopathogenesis of pneumonia, especially severe pneumonia disease. The intensity of these inflammatory responses often determines the severity of the disease (44). One study focused on pneumonia demonstrated that BTLA protein expression was mainly present in the bronchial epithelium and inflammatory cells in patients with severe community-acquired pneumonia (CAP), suggesting that BTLA might be involved in host protection. The percentages of circulating BTLA^+^CD4^+^ lymphocytes were significantly higher in patients with severe CAP and in mice with lipopolysaccharide (LPS)-induced acute lung inflammation than in control groups. Increasing BTLA expression *via* either the administration of dexamethasone or the agonistic anti-BTLA antibody 6A6 attenuates LPS-induced acute lung inflammation in mice (**Table 4**) (45). BTLA may be involved in regulating the immune response in patients with severe CAP, affecting the outcome of this disease.

**Table 4:** The function of BTLA in severe CAP and ARDS.

Study. Year	Specimen	Subjects	Main findings	Reference
Zhou et al., 2016	Peripheral blood BALF (only in mice) Mucosal biopsy specimens	11 patients with severe CAP 10 healthy controls Mice with LPS-induced acute lung inflammation Control mice	1. The percentages of circulating BTLA^+^CD4^+^ lymphocytes were significantly higher in patients with severe CAP and in mice with LPS-induced acute lung inflammation than in the control groups. 2. BTLA was mainly expressed in the bronchial epithelium and inflammatory cells.	(45)
Cheng et al., 2020	Lung	An ARDS group of rats A PBS control group of rats An ARDS + LRMSCs group of rats	1. The expression of BTLA was increased on the surface of alveolar macrophages (AMs) and pulmonary CD4^+^ lymphocytes of ARDS rats. 2. BTLA is involved in the immunoregulatory process operated by LRMSCs	(46)

ARDS, acute respiratory distress syndrome; BALF, bronchoalveolar lavage fluid; BTLA, B and T lymphocyte attenuator; CAP, community-acquired pneumonia; LPS, lipopolysaccharide; LRMSCs, lung-resident mesenchymal stem cells.

Severe pneumonia may cause acute respiratory distress syndrome (ARDS). ARDS is an organ failure syndrome caused by inappropriate and uncontrolled inflammatory response. The main pathophysiological feature of ARDS is inflammatory cytokine storm caused by overactivation of the immune system (47, 48). The expression of BTLA was increased on the surface of alveolar macrophages (AMs) and pulmonary CD4^+^ lymphocytes of ARDS rats (46). The treatment with lung-resident mesenchymal stem cells (LRMSCs), a promising candidate for ARDS therapy by regulating excessive inflammatory responses, can increase the expression of BTLA on immune cells. When BTLA expression was knockdown by siRNA, the immunoregulatory effects of LRMSCs were partially abolished, indicating that BTLA is involved in the immunoregulatory process operated by LRMSCs (**Table 4**) (46). Thus, BTLA may serve as a target for ARDS treatment.

## BTLA and Lung Cancer

The evasion of immune attack and formation of an immune suppressive environment within tumor exist during the entire process of cancer development (49). Immune checkpoint molecules can create immunosuppressive conditions in various cancers to impact tumorigenesis (50). Previous researches have shown the up-regulation of BTLA in gastric cancer (51), pancreatic cancer (52) and lymphocytic leukemia (53) and BTLA overexpression has been found to be associated with an immunosuppressive microenvironment (54). The blockade of the immunoinhibitory HVEM-BTLA/CD160 pathways may result in sustained tumor regression (55). One study found that the expression of BTLA on intratumoral CD8^+^ T cells was enhanced along with the progression of disease, and the co-expression of BTLA and other co-inhibitory molecules could inhibit T cell function (56). Similarly, the expression of BTLA was increased on CD4^+^ T cells and CD8^+^ T cells isolated from pleural effusion of lung cancer patients, indicating BTLA might mediate a negative cosignal for local immune response (57). Furthermore, BTLA was also expressed in tumor cells of non-small cell lung cancer (NSCLC) patients and the BTLA levels were significantly higher in patients with lymphatic metastasis and high tumor pathological stage (58). Those findings indicate that lung cancer can affect the body’s immune status through BTLA. A research in mice that received subcutaneous implantation of lung cancer cells showed that the expression of BTLA on CD4^+^ T cells and CD8^+^ T cells increased and the number of these T cells increased as well, while BTLA^+^/CD8^+^ T cells produced less IL-2 and TNF. The results indicated that tumor could induce enhanced expression of BTLA by T cells, impair T cell functions, thus led to systemic immunosuppression state (59). This may be the reason why tumor can escape from immune surveillance, and make tumor-bearing individuals more susceptible to infections (60). Moreover, a research showed that in lung adenocarcinoma that displayed an epithelial-mesenchymal transition (EMT) phenotype, BTLA expression was elevated in mesenchymal tissues, indicating that BTLA might influence the EMT of tumor by changing the inflammatory tumor microenvironment, then influence tumor metastasis and drug resistance (61). As for the impact on prognosis, Li et al. found high BTLA expression might predict the progression and poor prognosis of NSCLC. Patients with positive BTLA expression had a shorter relapse-free survival (RFS) than those with negative BTLA expression (58). A pharmacogenetic study suggested that a *BTLA* polymorphism with potential function to modify miRNA binding sites (rs76844316) was connected to the occurrence and prognosis of lung cancer (62). BTLA may be a novel therapeutic target for cancer immunotherapy (**Table 5**).

**Table 5:** The function of BTLA in lung cancer.

Study. Year	Specimen	Subjects	Main findings	Reference
Wang et al., 2006	Peripheral blood and pleural fluid	6 patients with lung cancer 6 healthy controls	The expression of BTLA was increased in the CD4^+^ and CD8^+^ T cells of pleural fluid of patients with lung cancer.	(57)
Thommen et al., 2015	Fresh tumor tissues and malignant effusions	32 patients with NSCLC	BTLA was generally expressed at a low percentage of tumor-infiltrating CD8^+^ T cells.	(56)
Mittal et al., 2015	Spleen	Mice that received subcutaneous implantation of lung cancer cells wild-type mice	1. The frequencies of BTLA^+^ cells in both the CD4^+^ and CD8^+^ T cell compartments were increased in mice with localized cancer relative to non-cancer controls. 2. BTLA^+^CD8^+^ T cells in cancer mice exhibited reduced IL-2 and TNF.	(59)
Lou et al., 2016	Paraffin-embedded tissues	439 patients with lung adenocarcinomas from three clinical datasets	BTLA were elevated in mesenchymal lung adenocarcinoma.	(61)
Li et al., 2020	Paraffin-embedded tissues	87 patients with stage I–III NSCLC	1. BTLA was expressed in tumor cells in 35 patients with NSCLC (40.2%). 2. BTLA levels were significantly higher in NSCLC patients with lymphatic metastasis and high tumor pathological stage. 3. Patients with positive BTLA expression had a shorter relapse-free survival (RFS) than those with negative BTLA expression.	(58)
Khadhraoui et al., 2020	Peripheral blood	169 patients with lung cancer 300 healthy controls	BTLA rs1982809 AG genotype carriers had a higher risk of developing lung cancer when compared to AA genotype carriers in Tunisian population.	(62)

BTLA, B and T lymphocyte attenuator; NSCLC, non-small cell lung cancer.

## Discussion

**Figure 1:**
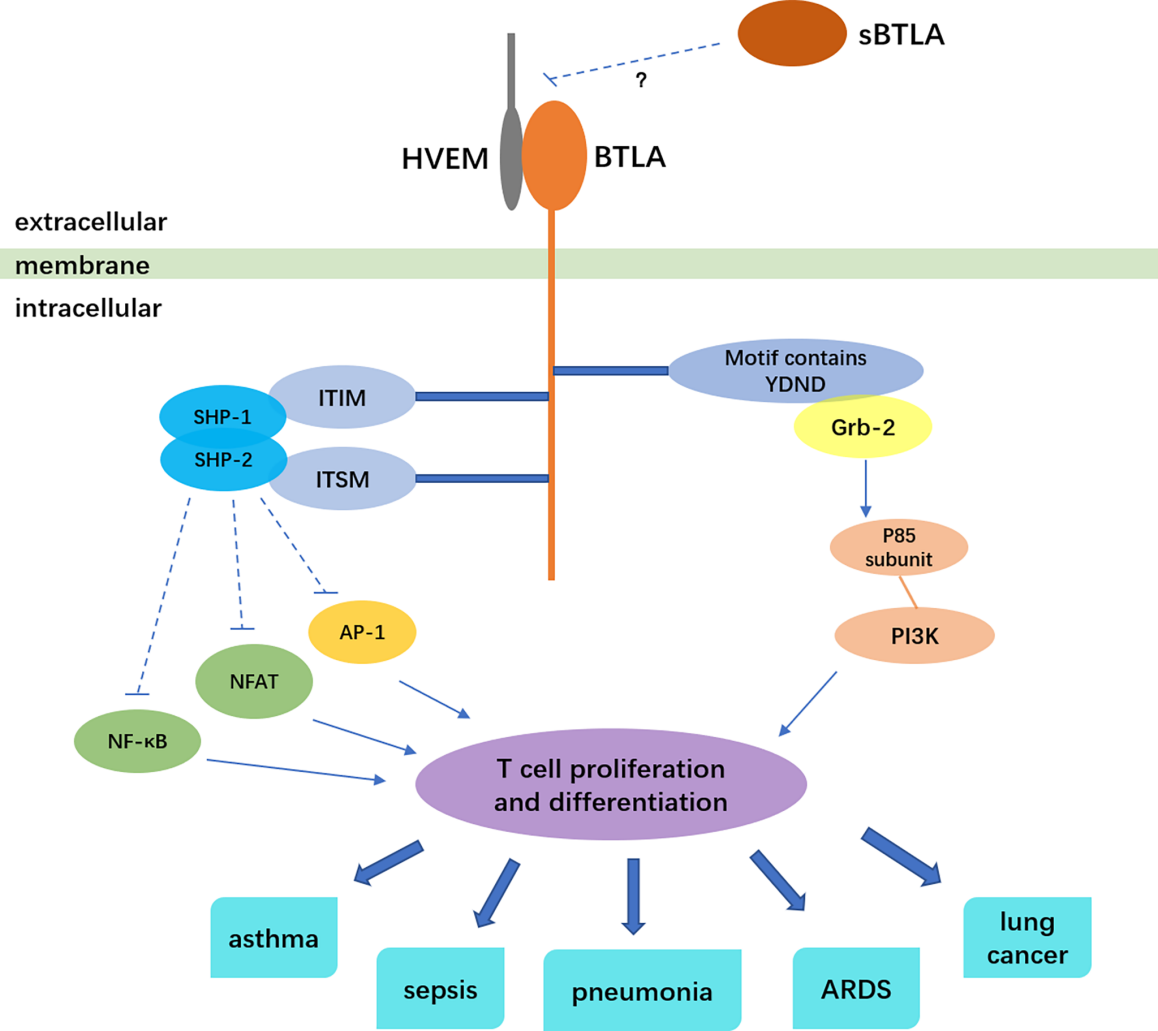
BTLA signaling and the relation to respiratory diseases. The solid arrows indicate stimulatory effect, the dotted lines indicate inhibitory effect.

BTLA plays an important role in immunoregulation and is involved in the pathogenesis of various respiratory diseases (**Figure 1**). In spite of its importance in regulating immunity, the HVEM-BTLA signaling in respiratory system diseases has not been sufficiently analyzed. One reason is that BTLA does not merely serve as an immune suppression role in respiratory system diseases. In many circumstances, BTLA can promote immunity and fight against infection. BTLA contains structure of promotive function (63), so it may produce immune enhancement signals during signal transduction. Besides, the binding of BTLA to its ligand HVEM can form a bi-directional signal system. BTLA and HVEM can act as ligand and receptor for each other, delivering different signals. When BTLA binds to HVEM as a ligand, it generates positive immune regulation (9, 64). For example, the BTLA involvement can induce HVEM-mediated NF-κB activation, which is important for the induction of pro-inflammatory and cell survival genes (65). In addition, BTLA lays in a complicated network of immune modulation and signal transmission. Researches separating one pathway from the network may not be comprehensive enough. Since HVEM and BTLA are widely expressed by many cell types, the exact regulatory mechanism in different immune contexts need to be carefully determined. So far, most researches merely find how the level of BTLA changes in different respiratory diseases. The underlying mechanism is still unknown. How HVEM-BTLA signaling regulates immunity and influences the pathogenesis of respiratory diseases need to be elucidated. More researches on mechanisms should be conducted. Based on current research, the level of BTLA may be used as an indicator of disease severity, and may predict the prognosis. Anti-BTLA antibody has been used in animal experiments to treat severe community-acquired pneumonia and epithelial ovarian carcinoma (45, 66). Antibodies in researches exert different effects, either agonistic or antagonistic (40, 67), which may due to HEVM-BTLA bi-directional signal system. An anti-BTLA monoclonal antibody has been approved for clinical trial by FDA (68). Treatment of respiratory diseases by anti-BTLA antibody must be on the road, more and more application researches will be conducted.

## Author Contributions

ZD and YZ reviewed the literature and drafted manuscript. PC edited and revised manuscript. ZZ was responsible for the conception. All authors contributed to the article and approved the submitted version.

## Funding

This work was supported by Research Project of Health Commission of Hunan Province (202103021536, 20200136, 20200121), National Natural Science Foundation of China (81903111), as well as Natural Science Foundation of Hunan Province (2020JJ8077).

## Conflict of Interest

The authors declare that the research was conducted in the absence of any commercial or financial relationships that could be construed as a potential conflict of interest.

## Abbreviations

Ams, alveolar macrophages; ARDS, acute respiratory distress syndrome; ATB, active pulmonary tuberculosis; APCs, antigen presenting cells; BTLA, B and T lymphocyte attenuator; CAP, community-acquired pneumonia; CMV, cytomegalovirus; CTLA-4, cytotoxic T lymphocyte antigen-4; DCs, dendritic cells; EMT, epithelial-mesenchymal transition; Grb-2, growth factor receptor binding 2; HVEM, herpes virus entry mediator; ITIMs, immunoreceptor tyrosine-based inhibitory motifs; LPS, lipopolysaccharide; LRMSCs, lung-resident mesenchymal stem cells; mDCs, myeloid dendritic cells; Mtb, mycobacterium tuberculosis; NSCLC, non-small cell lung cancer; PD-1, programmed death 1; RFS, relapse-free survival; RORγt, retinoid-related orphan receptor gamma-t; TB, tuberculosis; TNFR, tumor necrosis factor receptor.
